# Subjective Affective Responses to Natural Scenes Require Understanding, Not Spatial Frequency Bands

**DOI:** 10.3390/vision8020036

**Published:** 2024-06-04

**Authors:** Serena Mastria, Maurizio Codispoti, Virginia Tronelli, Andrea De Cesarei

**Affiliations:** Department of Psychology, University of Bologna, Alma Mater Studiorum, Viale Berti Pichat 5, 40127 Bologna, Italy; serena.mastria4@unibo.it (S.M.); maurizio.codispoti@unibo.it (M.C.); virginia.tronelli2@unibo.it (V.T.)

**Keywords:** emotion, spatial frequencies, scene understanding

## Abstract

It is debated whether emotional processing and response depend on semantic identification or are preferentially tied to specific information in natural scenes, such as global features or local details. The present study aimed to further examine the relationship between scene understanding and affective response while manipulating visual content. To this end, we presented affective and neutral natural scenes which were progressively band-filtered to contain global features (low spatial frequencies) or local details (high spatial frequencies) and assessed both affective response and scene understanding. We observed that, if scene content was correctly reported, subjective ratings of arousal and valence were modulated by the affective content of the scene, and this modulation was similar across spatial frequency bands. On the other hand, no affective modulation of subjective ratings was observed if picture content was not correctly reported. The present results indicate that subjective affective response requires content understanding, and it is not tied to a specific spatial frequency range.

## 1. Introduction

Our perceptual system can effectively identify relevant events in the environment that are potentially rewarding, or, otherwise, represent threats, in order to take appropriate action to assess, approach, or avoid them. When people are asked to describe the affective state elicited by pictorial cues that depict motivationally relevant content (e.g., depictions of mutilation, injuries, or nude bodies), the affective dimensions of valence (i.e., pleasant vs. unpleasant) and affective arousal (i.e., intensity; [1,2,3,4,5,6]) capture most of the variance in the reports, supporting the view that affective states are organized by a limited number of continuous dimensions [3,7,8,9,10]. When large samples of stimuli representing different contents were rated, the resulting distribution of affective states in the two-dimensional space was consistent with the motivational perspective, i.e., judgments of pleasantness indicate which motivational system is active, and judgments of arousal indicate the intensity of motivational activation [6].

In real life, objects or individuals are often viewed in crowded or cluttered conditions, from different viewpoints or distances, and our visual system is surprisingly efficient in recognizing them. Previous studies have shown that the viewing of emotionally arousing scenes (pleasant and unpleasant) modulates subjective, autonomic, and cortical changes even under perceptually challenging conditions. For instance, emotional engagement is observed when participants viewed natural scenes presented in small size [11,12], in the periphery of the visual field [13], or even for a very brief duration [14]. Similarly, some studies investigated how the compositional features of pictures modulate emotional response; in doing so, scene composition was operationalized as the result of the presence of global features that span most of a picture’s area (low spatial frequencies), local details that cover small portions of the image (high spatial frequencies), and an organizing structure that informs about where each detail is (phase of the spatial frequency spectrum) [15]. When manipulating the compositional structure of natural scenes, i.e., by filtering pictures to contain only global or local information [16,17], or by scrambling the phase of the spatial frequency spectrum [18], it was observed that even filtered or phase-scrambled scenes can elicit an affective response. At the same time, the abovementioned studies demonstrated that the various indexes of emotional response (subjective, autonomic, and central) were modulated only if participants could discriminate among emotional picture contents. If participants could not categorize or discriminate picture content, no affective modulation of behavioral, cortical, autonomic, or subjective response was observed (“semantic primacy” hypothesis; [19,20]).

Several findings indicate that scene content understanding is a necessary condition for the engagement of appetitive and defensive motivational systems in the context of natural scene perception [18,21,22]. In particular, when the phase of the spatial frequency spectrum was scrambled, accuracy in a human/animal task dropped to chance level [18]; importantly, when phase scrambling was reduced, it was not until classification accuracy increased to over 80% that emotional pictures could elicit a modulation of cortical responses (Late Positive Potential, LPP; [4,23,24,25,26]). In a task prompting participants to view pictures in progressively less degraded versions and deciding when they had got the gist of the scene, LPP modulation was first observed beginning one step before the gist, suggesting a strong link between the decisional process involved in stimulus identification and the affective modulation of the LPP [21]. Moreover, in a paradigm that compared the speed of response to several categorization tasks on the same emotional and neutral pictures, affective judgements were shown to have greater latencies compared with basic, superordinate, and subordinate categorization [27]. Consistently, chronometrically examining the speed of semantic vs. affective judgments, it was observed that semantic judgments preceded affective ones by 120 ms [20]. Altogether, these results support the view that identification is a conditio sine qua non for affective response (e.g., [14,18,20,21,22,27]). 

On the other hand, some studies suggest that the emotional content of the pictures may influence subjective emotional judgments even when participants do not achieve semantic understanding of scene content, suggesting that the elicitation of an affective experience operates independently of identification. Results supporting this view often come from studies employing backward masking, in which the temporal visual availability of a stimulus is limited and the retinal persistence is eliminated by the presentation of a visually salient image immediately after the presented scene [28,29]. However, results from subliminal presentation often failed to be replicated [30,31,32]. Other studies manipulated spatial frequency content and suggested that emotional responses are consequential to the analysis of low spatial frequencies, which is carried out independently of conscious analysis and identification [33,34] However, more pronounced responses at specific spatial frequency ranges can be consequential either to a preferential processing of affective contents at a specific spatial frequency range, or to a better identification. To disentangle these two possibilities, it is necessary to balance the processing efficiency of these frequency ranges. For instance, when stimuli are high- or low-pass filtered, an identification task can be used to inform on the semantic understanding of the presented scene. However, few studies to date have adopted this strategy [21,35,36,37]. 

What is the contribution of visual content and of scene understanding to the subjective affective response to natural images? Does affective response require semantic content identification to be elicited, or are they independent? The present study aimed to further examine the relationship between scene understanding and affective response while manipulating visual content. To this end, we created pictures that only contained specific, band-passed spatial frequencies (i.e., visual features of a certain range) and that could represent emotional (pleasant or unpleasant) or neutral contents. In terms of affective response, we focused on the subjective reports of affective arousal and hedonic valence. Moreover, after each picture, we asked participants to report in an open description what they had identified in a scene (e.g., “a child playing with a ball”). The accuracy of these open descriptions was used to assess, independently of the spatial frequency presented, the semantic identification of natural scenes. If visual content is critical for affective response but identification is not, it is expected that the scene content and spatial bandpass filter will interact in modulating affective ratings of arousal and valence, regardless of semantic identification. On the other hand, if identification is critical, one might expect that the modulation of affective response is only observed when participants correctly report the content of the scene. 

As a second aim, we were interested in the possible preferential role for low compared with high spatial frequencies. It has been speculated that low spatial frequencies (coarsest features of a scene) might be sufficient to elicit emotional responses [33,34,38,39,40]. Therefore, building on this hypothesis, the affective reaction to scenes that are presented in their coarsest details should be more pronounced, or more resistant to lack of identification, compared with scenes that are presented in their finest details. On the other hand, if identification is a critical factor for affective response, then similar effects should be observed for high and low spatial frequencies once equated for identification. 

## 2. Method

### 2.1. Participants

A total of 15 participants took part in the present study (12 females, mean age = 22.06, SD = 1.39). The sample size was determined on the basis of a previous pilot sample of participants using G*Power 3.1 [41], with the parameters alpha = 0.05, power = 0.80, partial eta squared = 0.06 (medium effect according to [42]), and correlation among repeated measures = 0.75 to detect an interaction between spatial frequency band (5 levels) and picture content (3 levels). The power analysis indicated a minimum sample size of 8 participants. All participants had self-reported normal or corrected-to-normal visual acuity, and none of them reported current or past neurological or psychopathological problems. Informed consent was obtained from all participants. The experimental protocol conforms to the Declaration of Helsinki and was approved by the Ethical Committee of the University of Bologna.

### 2.2. Stimuli and Equipment

We selected a total of 90 pictures from various sources, including the International Affective Picture System (IAPS; [43]) and public-domain pictures available on the Internet. The following picture categories were selected: erotic couples (arousing, pleasant, N = 15); babies (arousing, pleasant, N = 15); indoor or outdoor people in daily activities (neutral, N = 30); attacks (arousing, unpleasant, N = 15); and injuries (arousing, unpleasant, N = 15). These pictures were homogeneous with respect to brightness and contrast (pixel intensity M = 128, SD = 72.11), non-blurriness of the foreground and background, number of portrayed people (between 1 and 3 persons per picture, with visible face), and picture area covered by the face of a single individual (percent of total picture size M = 10.76%, SD = 3.41%, to discard pictures in which people were too close or too far away from the viewer). 

**Figure 1:**
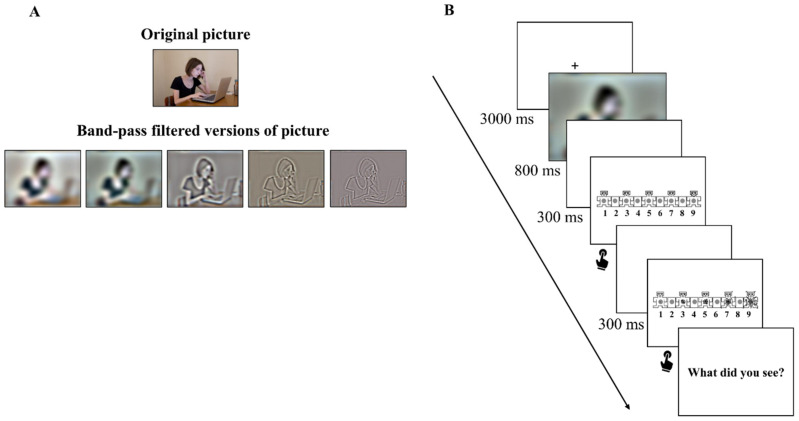
(**A**) Original (on the top) and band-passed (on the bottom) versions of one sample picture. The image shown in the picture is not part of the experimental database and is ©University of Bologna licensed for research use. Filtering levels were adjusted to the printed version of the picture; (**B**) Procedure of each trial. After viewing each picture, participants rated their affective state of valence and arousal on a 1–9 scale. Next, they were asked to describe the gist of the scene.

Each picture was filtered using a band-passed filter which was centered around the following spatial frequencies, which will be defined as F_0_: 4, 13.5, 45.3, 152.2, 512 cpi. The bandpass filter was implemented as the combination of a low-pass and a high-pass filter, which passed all frequencies up to F_0_ and reached zero at F_0_·3 for the low-pass filter, and passed all frequencies from the highest frequency down to F_0_ and reached zero at F_0_/3 for the high-pass filter. An example of degraded pictures participants viewed in the experiment is displayed in Figure 1A, showing the non-degraded version on the top. Each of the 90 pictures was degraded in 5 different bands of degradation. Each participant saw each image once in one type of degradation. The images were 21 × 17 cm in size, with a visual angle of 23.72° horizontal × 19.30° vertical. Pictures were presented on a 17-inch monitor, from a distance of 50 cm, using Open Sesame software, version 3.2.7 Kafkaesque Koffka [44].

### 2.3. Procedure

The experiment took place inside a dimly lit laboratory room. The experimental procedure consisted of 90 trials, preceded by 4 practice trials. As depicted in Figure 1B, during each trial, a fixation cross was presented for 3000 ms. After this time, an image was displayed and remained visible for 800 ms. The visual rating scales of valence and arousal were presented using the Self-Assessment Manikin (SAM; [45,46]); each scale was preceded by a blank interval lasting 300 ms. Participants were asked to rate the affective state elicited by each picture on a 9-point scale on the dimension of affective valence and arousal. Finally, participants were asked to describe the gist of the scene by typing a short answer. The whole experimental procedure took approximately 60 min.

### 2.4. Data Analysis

Practice trials were excluded from the analysis. Accuracy scoring was carried out manually for each participant. The answers to the question “what did you see?” were analyzed with the following method: we classified each answer into one of the categories which were present in the study (erotic couples, babies, neutral people, attacks, and mutilation). A response was scored as accurate if the scored category matched the picture category. Two raters independently evaluated the accuracy of each response without being aware of the filtering condition that was applied. The inter-rater agreement calculated on the total number of the responses was high (Cohen’s κ = 0.917). Accuracy data were submitted to repeated-measures ANOVAs with Band (five levels: 4, 13.5, 45.3, 152.2, and 512 cpi) and Content (three levels: pleasant, neutral, and unpleasant) as the within-subject factors. If a superordinate main effect or interaction was significant, we carried out separate ANOVAs on subordinate conditions or post hoc comparisons. Greenhouse–Geisser correction was applied when sphericity assumption was not met. The partial eta squared statistic (η^2^_p_), indicating the proportion between the variance explained by one experimental factor and the total variance, was calculated and reported. 

Arousal and valence ratings were analyzed across filtering bands, content, and accuracy in the content description task. As picture degradation was minimum for intermediate levels (see Figure 1A), accuracy was unequally distributed, and missing cells in the ANOVA design were observed for non-accurate trials in intermediate spatial frequency bands and, to a lesser extent, for accurate trials in the lowest and highest spatial frequency bands. To deal with these missing cells, we first replaced missing values using the multiple imputation method [47] using Amelia II [48] software, with Participant as cross-section variable, Degradation and Content as nominal variables, and arousal and valence as continuous variables which were constrained in the 1–9 range. A total of 5 different datasets were produced and analyzed, and F values were pooled from the analysis of these datasets. The *p* and partial eta squared values were recalculated for the pooled F values using the original degrees of freedom. Finally, a control analysis without missing data replacement was carried out which, although with smaller statistical power, confirmed the same pattern of results. 

## 3. Results

### 3.1. Identification Performance

**Figure 2:**
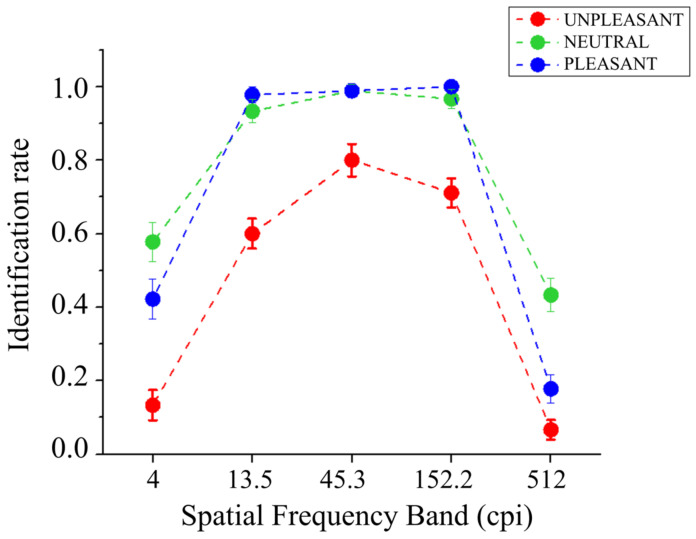
Identification rate (from 0 or inaccurate to 1 or accurate) of degraded natural scenes as a function of emotional content and the five spatial frequency bands.

The identification performance of bandpass-filtered pictures as a function of picture content is reported in Figure 2. A significant main effect of Band was found (*F*_(4,56)_ = 316.71, *p* < 0.001, and *η*^2^_p_ = 0.96), showing better identification in the intermediate bands (13.5, 45.3, and 152.2 cpi), compared with the lowest (4 cpi) and the highest (512 cpi) bands, and better identification for the lowest compared with the highest band. Significant differences were observed between all bands (*F*s_(1,14)_ > 62.93, *p*s < 0.001, and *η*^2^*_p_*s > 0.82), except between the third (45.3 cpi) and the fourth (152.2 cpi) bands (*F*_(1,14)_ = 3.86, *p* = 0.070, and *η*^2^_p_ = 0.22). A significant main effect of Content was also observed (*F*_(2,28)_ = 61.07, *p* < 0.001, and *η*^2^_p_ = 0.81), indicating less accurate identification for unpleasant pictures compared with both pleasant (*F*_(1,14)_ = 76.53, *p* < 0.001, and *η*^2^_p_ = 0.85) and neutral scenes (*F*_(1,14)_ = 116.57, *p* < 0.001, and *η*^2^_p_ = 0.89), and the latter two picture contents did not significantly differ from each other (*F*_(1,14)_ = 4.16, *p* = 0.061, and *η*^2^_p_ = 0.23). The Band by Content interaction was significant (*F*_(8,112)_ = 5.06, *p* = 0.001, and *η*^2^_p_ = 0.27). Following this significant interaction and focusing on each spatial frequency band, a significant effect of picture content was observed in all bands (*F*s_(2,28)_ > 15.21, *p*s < 0.001, and *η*^2^*_p_*s > 0.52). Specifically, a significantly more accurate identification for both neutral and pleasant pictures compared with unpleasant ones was observed in bands from the first (4 cpi) to the fourth (152.2 cpi) (*F*s_(1,14)_ > 11.92, *p*s < 0.004, and *η*^2^*_p_*s > 0.46), with no differences between neutral and pleasant contents (*F*s_(1,14)_ < 4.07, *p*s > 0.063, and *η*^2^_p_s < 0.22). In the highest band (512 cpi), neutral pictures were identified better than both pleasant and unpleasant pictures (*F*s_(1,14)_ > 12.43, *p*s < 0.01, and *η*^2^*_p_*s > 0.47); the latter were significantly different from each other (*F*_(2,28)_ = 8.232, *p* = 0.012, and *η*^2^_p_ = 0.37), with higher identification for pleasant pictures than unpleasant ones.

### 3.2. Affective Response

Affective ratings were analyzed as a function of scene identification. As scene identification was extremely accurate in the intermediate frequency bands for most participants, it was not possible to have a fully balanced ANOVA design. While the multiple imputation method allowed us to replace missing values, the extremely high number of missing cells in non-accurate intermediate bands (up to 100%, with an average of more than 75% missing cells across participants in one content condition, with several of these participants only providing one trial per condition; see Table 1) suggested that the non-missing data in these intermediate levels might not suffice for missing data replacement, and they were therefore dropped from the analysis. For this reason, we first analyzed subjective affective reactions to scenes that were accurately described using an ANOVA design with factors Band (five levels) and Content (three levels). Then, we analyzed the effects of accuracy on the subjective ratings of arousal and valence to the most extreme spatial frequency bands using an ANOVA with factors Accuracy (accurate and inaccurate), Band (lowest and highest), and Content (pleasant, neutral, and unpleasant).

#### 3.2.1. Arousal Ratings

**Figure 3:**
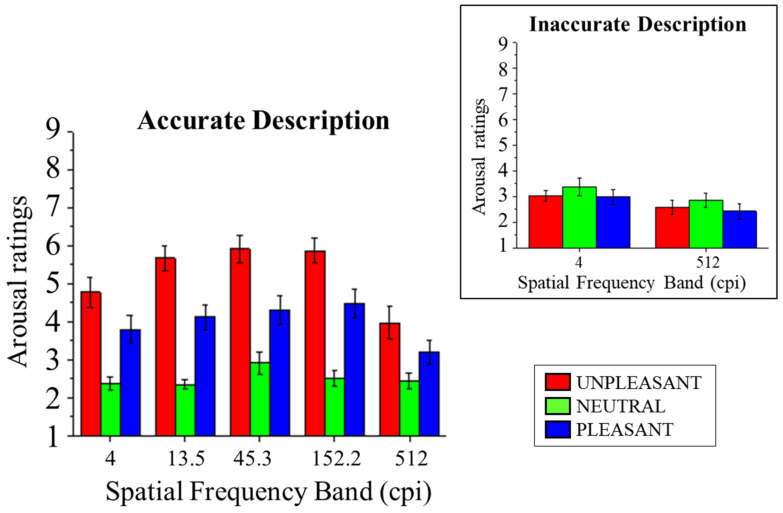
SAM ratings of arousal for pleasant, neutral, and unpleasant pictures as a function of the five spatial frequency bands when scene identification was achieved. The inset shows arousal scores in the lowest (4 cpi) and the highest (512 cpi) spatial frequency band as a function of emotional content when scene identification was not achieved. Error bars represent the within-participant standard error of the mean [49].

Subjective affective ratings of arousal are presented in Figure 3. In accurate trials, a significant main effect of Content was observed (*F*_(2,28)_ = 39.10, *p* < 0.001, and *η*^2^_p_ = 0.74), indicating that affective responses to both unpleasant and pleasant pictures were rated as more arousing than neutral pictures (*F*s_(1,14)_ > 27.90, *p*s < 0.001, and *η*^2^_p_s > 0.67) and that arousal ratings were higher following unpleasant compared with pleasant contents (*F*_(1,14)_ = 11.70, *p* = 0.004, and *η*^2^_p_ = 0.46). 

A significant interaction of Band and Content was observed (*F*_(8,112)_ = 2.50, *p* = 0.016, and *η*^2^_p_ = 0.15). Following this significant interaction, the effects of Content were assessed in each band for accurate trials. In all bands, a significant effect of Content was observed (*F*s_(2,28)_ > 6.23, *p*s < 0.006, and *η*^2^_p_s > 0.31), with higher arousal ratings for unpleasant and pleasant compared with neutral scenes (*F*s_(1,14)_ > 10.98, *p*s < 0.005, and *η*^2^_p_s > 0.44), with the exception of pleasant vs. neutral in the highest band (*F*_(1,14)_ = 3.94, *p* = 0.067, and *η*^2^_p_ = 0.22). Unpleasant scenes were rated as more arousing than pleasant scenes in the three intermediate levels (*F*s_(1,14)_ > 6.314, *p*s < 0.025, and *η*^2^_p_s > 0.31). Finally, a significant effect of Band was observed (*F*_(4,56)_= 9.39, *p* < 0.001, and *η*^2^_p_ = 0.40), with lower ratings for scenes in the lowest and highest spatial frequency bands compared with all other spatial frequency bands (*F*s_(1,14)_ > 6.51, *p*s < 0.023, and *η*^2^_p_s > 0.32) and lower ratings for scenes in the highest than in the lowest band (*F*_(1,14)_ = 8.16, *p* = 0.013, and *η*^2^_p_ = 0.37). 

Directly focusing on the effects of identification accuracy on affective ratings in challenging conditions, i.e., the lowest and highest spatial frequency bands, we observed a significant interaction between Content and Accuracy (*F*_(2,28)_ = 12.27, *p* < 0.001, and *η*^2^_p_ = 0.47), indicating that no significant effect of Content was observed for non-accurate trials (*F*_(2,28)_ = 2.15, *p* = 0.135, and *η*^2^_p_ = 0.13), while it was observed for correctly described scenes (*F*_(2,28)_ = 13.19, *p* < 0.001, and *η*^2^_p_ = 0.49), with higher arousal ratings for pleasant and unpleasant compared with neutral scenes (*F*s_(1,14)_ > 11.46, *p*s < 0.004, and *η*^2^_p_s > 0.45), and no significant difference between ratings for pleasant and unpleasant scenes (*F*_(1,14)_ = 3.87, *p* = 0.069, and *η*^2^_p_ = 0.22). No significant three-way interaction between Content, Accuracy, and Band was observed (*F*_(2,28)_ = 0.99, *p* = 0.386, and *η*^2^_p_ = 0.07), indicating that the spatial frequency band (lowest vs. highest) did not add to the effects of accuracy and content. Overall, a significant main effect of Content was observed (*F*_(2,28)_ = 8.55, *p* = 0.001, and *η*^2^_p_ = 0.38), indicating higher arousal ratings for unpleasant than neutral and pleasant scenes (*F*s_(1,14)_ > 5.39, *p*s < 0.036, and *η*^2^_p_s > 0.28) but not for pleasant compared with neutral scenes (*F*_(1,14)_ = 3.41, *p* = 0.086, and *η*^2^_p_ = 0.20). Finally, a significant effect of Accuracy was observed (*F*_(1,14)_ = 6.06, *p* = 0.027, and *η*^2^_p_ = 0.30), with higher ratings for correctly compared with incorrectly described scenes, and for Band (*F*_(1,14)_ = 8.16, *p* = 0.013, and *η*^2^_p_ = 0.37), with higher ratings for the lowest compared with the highest spatial frequency band.

#### 3.2.2. Valence Ratings

**Figure 4:**
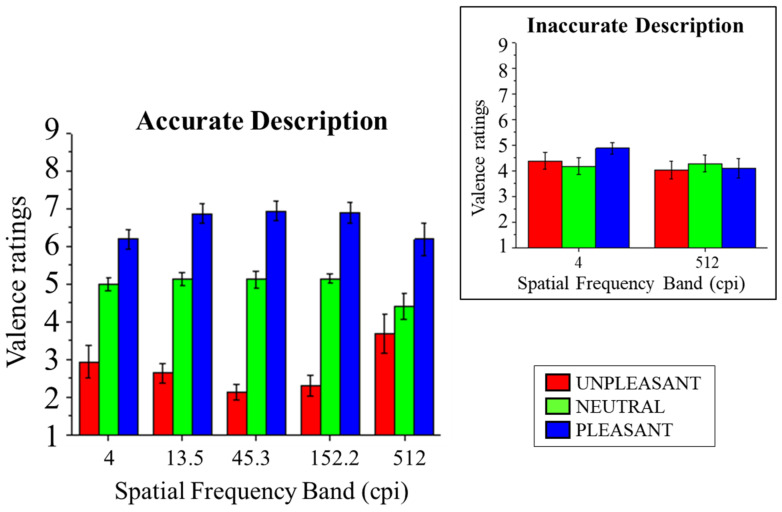
SAM ratings of valence for pleasant, neutral, and unpleasant pictures as a function of the five spatial frequency bands when scene identification was achieved. The inset shows valence scores in the lowest (4 cpi) and the highest (512 cpi) spatial frequency band as a function of emotional content when scene identification was not achieved. Error bars represent within-participant standard error of the mean.

Subjective affective ratings of valence are presented in Figure 4. In accurate trials, a significant main effect of Content was observed (*F*_(2,28)_ = 108.38, *p* < 0.001, and *η*^2^_p_ = 0.89), indicating a linear effect of Content on valence ratings, with lower ratings following unpleasant scenes compared with neutral (*F*_(1,14)_ = 110.54, *p* < 0.001, and *η*^2^_p_ = 0.89) and following neutral compared with pleasant scenes (*F*_(1,14)_ = 35.87, *p* < 0.001, and *η*^2^_p_ = 0.72). 

A significant interaction of Band and Content was observed (*F*_(8,112)_ = 4.01, *p* < 0.001, and *η*^2^_p_ = 0.22). Following this significant interaction, the effects of Content were assessed at each band for accurate trials. In all bands, a significant effect of Content was observed (*F*s_(2,28)_ > 7.80, *p*s < 0.002, and *η*^2^_p_s > 0.36), with linearly increasing ratings of valence according to the pleasantness of the scenes, unpleasant vs. neutral (*F*s_(1,14)_ > 18.34, *p*s < 0.001, and *η*^2^_p_s > 0.57) and neutral vs. pleasant (*Fs*_(1,14)_ > 7.45, *ps* < 0.016, and *η*^2^_p_s > 0.35), with the exception of unpleasant vs. neutral in the highest band (*F*_(1,14)_ = 1.02, *p* = 0.329, and *η*^2^_p_ = 0.07). No significant effect of Band was observed (*F*_(4,56)_= 0.42, *p* = 0.79, and *η*^2^_p_ = 0.03). 

An ANOVA with the factors Accuracy (accurate and inaccurate), Band (lowest and highest), and Content (pleasant, neutral, and unpleasant) was carried out on valence ratings. Scene Content interacted with Accuracy (*F*_(2,28)_ = 13.47, *p* < 0.001, and *η*^2^_p_ = 0.49), indicating that no significant effect of Content was observed for non-accurate trials (*F*_(2,28)_ = 1.16, *p* = 0.33, and *η*^2^_p_ = 0.08), while it was observed for correctly described scenes (*F*_(2,28)_ = 23.15, *p* < 0.001, and *η*^2^_p_ = 0.62), with lower valence ratings for unpleasant compared with neutral scenes (*F*_(1,14)_ = 11.91, *p* = 0.004, and *η*^2^_p_ = 0.46) and for neutral compared with pleasant scenes (*F*_(1,14)_ = 15.73, *p* = 0.001, and *η*^2^_p_ = 0.53). No significant three-way interaction between Content, Accuracy, and Band was observed (*F*_(2,28)_= 2.45, *p* = 0.105, and *η*^2^_p_ = 0.15), indicating that the joint effects of Accuracy and Content did not differ between spatial frequency bands (lowest vs. highest). Finally, a significant main effect of Content was observed (*F*_(2,28)_ = 25.54, *p* < 0.001, and *η*^2^_p_ = 0.65), indicating linearly higher ratings of valence with increasing pleasantness of the scenes, unpleasant vs. neutral (*F*_(1,14)_ = 10.78, *p* = 0.005, and *η*^2^_p_ = 0.43) and neutral vs. pleasant (*F*_(1,14)_ = 18.05, *p* = 0.001, and *η*^2^_p_ = 0.56).

## 4. Discussion

Based on previous results in the picture-viewing context, in the present study, we investigated the relationship between scene identification and subjective emotional response, by manipulating the spatial frequency range of emotional and neutral scenes. The results showed that the ratings of arousal and valence were only modulated by the emotional content of the picture when scene content was correctly reported, regardless of the specific spatial frequency manipulation. No affective modulation of subjective ratings was observed when scene content was not correctly reported. 

Motivationally relevant cues prompt a broad range of emotional responses, involving subjective, autonomic, facial, and cortical changes [1,5,50,51,52] that reflect the engagement of motivational systems, one appetitive and one defensive, that have evolved to adaptively regulate human behavior in the environment [1,6,53,54,55]. These responses can be elicited even under perceptually degraded conditions, such as long distance, peripheral vision, or short exposure time, providing evidence that individuals can efficiently discriminate among emotional picture contents. An example of a perceptually degraded condition is the case of images that only allow the observer to perceive their global features (low spatial frequencies) or local details (high spatial frequencies). Studies that directly examined the relation between spatial frequencies and emotional response reported that spatially filtered versions of emotional scenes may still elicit emotional responses (e.g., [16,18,21,56]). Although some studies suggested that pictures that could not be identified may still elicit emotional responses (e.g., [28,29]), the present study is consistent with previous results in indicating that emotional reactions require stimulus identification [14,18,20,21,22,27]. 

Several studies investigating the relationship between identification and emotional response assessed identification using gist understanding or closed-category categorization [18,21,27]. Here, we used a conservative measure of scene identification and asked participants to write a concise description of the scene. In terms of spatial frequency ranges, we observed that identification for all contents was higher in the intermediate frequency bands, supporting the view that no picture content relied critically on extremely low or high spatial frequency bands for identification [21,22,57,58]. In line with previous findings, we also observed that unpleasant scenes that included injuries and mutilated bodies were particularly difficult to understand and report compared with other contents [18,21]. This result suggests that discrimination difficulty is dissimilarly distributed among scene contents, possibly due to the different compositional features that are characteristic of each category. For instance, specific difficulties in understanding unpleasant pictures can come from the sometimes unusual positions of injured people or to missing body parts in some pictures. In terms of the idiosyncratic effects of each picture, future studies may use larger samples of pictures and attempt to pinpoint specific regularities (e.g., in terms of the presence of a distinctive feature or of sub-categories) that are responsible for category-specific effects. However, despite the fact that discrimination difficulty was higher for unpleasant scenes and for the highest and lower ends of the spatial frequency spectrum, affective responses to correctly described scenes were remarkably similar as a function of the spatial frequency range. In this respect, the only change in affective response was observed in the highest spatial frequency band (around 512 cpi), in which arousal ratings for pleasant and neutral scenes did not significantly differ from each other, and no significant difference between valence ratings for unpleasant and neutral pictures was observed. This result is consistent with the observation that affective categorization follows semantic categorization [27] and suggests that, although the content of these scenes was correctly described, the emotional modulation of affective states elicited by these scenes was less pronounced or absent. 

Here, we observed that emotional responses, assessed using ratings of valence and arousal, did not depend on the compositional features of the natural scenes, operationalized here as the spatial frequency range. Rather, a similar affective response in terms of rated valence and arousal was observed in all visual conditions once the scene content was correctly reported. As subjective ratings represent one facet of emotional response, and responses to emotional stimuli are also expressed in several cortical and autonomic activities and modulations, it may be asked whether other components of emotional responses are modulated by spatial frequency or by non-identified contents. In this respect, previous studies observed the affective modulation of autonomic and cortical responses only if the subjective modulation of affective ratings was achieved [14]. For instance, a study parametrically manipulated the exposure time of masked and unmasked stimuli from 25 ms to 6 s and failed to observe any affective modulation of the central, facial, and autonomic components of emotional response in the absence of the modulation of affective ratings. In the same study, participants who did not differentiate among pleasant and unpleasant contents in terms of rated pleasure showed no modulation as a function of picture content in other components of emotional response (e.g., cortical, facial, and electrodermal changes; [14]). 

Along with previous studies, we observed a similar modulation of affective judgments once pictures were recognized [14,18,20,21,22,27]. Based on the observation that the amount of perceptual details is linearly related to the distance between an observer and an object [59], several studies investigated the pattern of emotional response to objects varying in distance, detail, or looming behavior [56,60,61]. These studies indicated that, while some components of the emotional response (e.g., modulation of electrodermal activity) are highly sensitive to the imminence of an object, other components are less modulated by picture imminence and are almost or exclusively sensitive to the content of a picture [21,56,60]. As the context in which events happen may be relevant for some components of the emotional response, future studies might manipulate picture context through perceptual manipulations as in the present study (e.g., using a perceptual manipulation as a proxy for a real-life property, i.e., spatial frequency for distance), through other contextual properties (e.g., history of the most recently viewed pictures, e.g., [62,63,64]), or through artificially created scenarios, i.e., in virtual-reality simulations, in which emotional responses might be elicited and measured [65,66]. Provided that emotional events are correctly detected and identified [14,18,20,21,22,27], it is possible that sensitivity to contextual manipulations varies between components of the emotional response (e.g., autonomic sympathetic activation vs. central activity). 

As a secondary aim, we were interested in whether emotional responses rely on the semantic understanding of picture content (e.g., [18,21,22]) or are preferentially guided by specific visual information in natural scenes, such as low spatial frequencies [33,34,38,39,40]. In this respect, a preferential role of low spatial frequencies would have predicted that emotional responses to blurred stimuli would have been larger or less affected by the identification rate. However, neither prediction was observed, and no affective modulation was observed for either spatial frequency range when scene contents were not correctly reported. Moreover, when the combined effects of accuracy and of content were compared across spatial frequency bands, no difference between the two spatial frequency ranges was observed neither for arousal nor for valence ratings. Therefore, the current data do not support the view that a specific spatial frequency range (either low or high) contains information that is sufficient to elicit a subjective emotional response, which instead requires semantic content understanding. These results extend previous findings which showed that the emotional response, as indexed at the cortical level by the modulation of the LPP, did not depend on the compositional features of the natural scenes but instead varied with the identification of the affective content of the pictures, regardless of the spatial frequency range of natural scenes [21], and are consistent with the “semantic primacy” view of affective responses [19,20]. 

## 5. Conclusions

The present results are consistent with previous research in indicating that identification is a *conditio sine qua non* for affective response and that no isolated perceptual property or preference guides emotional response. When the emotional content of natural scenes can be recognized, it modulates evaluative judgments of pleasantness and arousal and results in the engagement of motivational systems.

## Figures and Tables

**Table 1:** Number of images correctly and incorrectly identified, for each emotional content and spatial frequency threshold.

Emotional Content				
Spatial Frequency Band	Description Accuracy	Unpleasant	Neutral	Pleasant
Lowest (4 cpi)	Inaccurate	78	38	52
	Accurate	12	52	38
13.5 cpi	Inaccurate	36	6	2
	Accurate	54	84	88
54.3 cpi	Inaccurate	18	1	1
	Accurate	72	89	89
152.2 cpi	Inaccurate	26	3	0
	Accurate	64	87	90
Highest (512 cpi)	Inaccurate	84	51	74
	Accurate	6	39	16

## Data Availability

Data is contained within the article and can be accessed upon request to the Corresponding Author.
